# Short- and long-term weather prediction based on a hybrid of CEEMDAN, LMD, and ANN

**DOI:** 10.1371/journal.pone.0304754

**Published:** 2024-07-22

**Authors:** Samuel Asante Gyamerah, Victor Owusu

**Affiliations:** 1 Department of Statistics and Actuarial Science, Kwame Nkrumah University of Science and Technology, Kumasi, Ghana; 2 Department of Agricultural Economics, Agribusiness and Extension, Kwame Nkrumah University of Science and Technology, Kumasi, Ghana; Abu Dhabi University, UNITED ARAB EMIRATES

## Abstract

Agriculture is one of the major economic sectors in Africa, and it predominantly depends on the climate. However, extreme climate changes do have a negative impact on agricultural production. The damage resulting from extreme climate change can be mitigated if farmers have access to accurate weather forecasts, which can enable them to make the necessary adjustments to their farming practices. To improve weather prediction amidst extreme climate change, we propose a novel prediction model based on a hybrid of complete ensemble empirical mode decomposition with adaptive noise (CEEMDAN), local mean decomposition (LMD), and artificial neural networks (NN). A detailed comparison of the performance metrics for the short- and long-term prediction results with other prediction models reveals that the three-phase hybrid CEEMDAN-LMD-NN model is optimal in terms of the evaluation metrics used. The study’s findings demonstrate the efficiency of the three-phase hybrid CEEMDAN-LMD-NN prediction model in decision-system design, particularly for large-scale commercial farmers, small-holder farmers, and the agricultural index insurance industry that require reliable forecasts generated at multi-step horizons.

## Introduction

The agriculture and agribusiness sectors account for a significant share of all the major economic activities and sources of income among smallholder farmers in most underdeveloped and developing countries. However, there has been an increase in the volatility of agriculture production (crop and livestock yields) due to extreme weather events, high climate variability, and hydrological flows [1]. Generally, weather is difficult to control, especially for small-holder farmers, and does usually have a great impact on their farming activities. For this reason, an effective and reliable weather risk management tool (Example, weather index insurance or weather derivatives) is needed to hedge farmers and agricultural stakeholders from the perils of weather variability and uncertainties [2–5]. Weather index insurance provides immense benefits to the agricultural sector. For instance, there is no problem with systemic risk in weather index insurance contracts since such contracts are designed to allow effective risk transfer instead of risk pooling. Farm level loss adjustments are reduced since the payoffs of the contract are linked to a weather index. Consequently, transaction costs are minimized in these contracts as compared to traditional crop insurance. However, the design of weather index insurance/weather derivatives is linked to an accurate forecast of the weather. The ability to accurately forecast the weather multiple steps ahead at a specific location is an essential component of both the demand and supply sides of the market for weather index insurance/weather derivatives. A reliable multiple-step weather forecast is important as it helps to determine future weather conditions for the demand and supply sides of the market for weather index insurance. Weather prediction helps in determining when or not to sow, irrigate, apply fertilizer, and start harvesting. However, with the increasing changes in climate, the reliability of weather prediction will be challenged, and the uncertain nature of weather implies that all forecasts are inherently uncertain [6]. Furthermore, the properties of weather (continuous, data-intensive, multidimensional, dynamic, and chaotic) make weather prediction a complex process. This increases the computational cost of weather prediction and poses challenging problems that require contributions from different experts in data science, information science, physics, climate science, and cognitive science.

However, in the last decade, it has been shown that machine learning techniques [7–10] are very robust and efficient in predicting weather variables such as temperature, rainfall, dewpoint, solar radiation, and wind, which are often complex and chaotic and can sometimes record extreme values [11]. Nonetheless, using only a machine learning model does not give accurate forecasting due to the seasonal, non-linear, volatile, and non-stationary [12] characteristics of most weather time series data. To overcome these characteristics and also to improve the forecasting accuracy of temperature forecasts, different studies [12–15] have proposed using a signal decomposition technique such as mode decomposition. The mode decomposition technique decomposes the non-stationary temperature data into relatively stationary and regular sub-series, which can accurately reflect the local features of the original temperature data series. The theory and concept of mode decomposition were first introduced by [16] and were called “Empirical Mode Decomposition (EMD)”. EMD is the first part of the Hilbert-Huang transform (HHT) and can deal with the volatile, non-linear, and non-stationary behaviour of weather data. It is, therefore, able to decompose weather time series data into different intrinsic mode functions (IMFs) by the process of sifting without leaving the time domain. The advantage of EMD is that the basic function is obtained from the signal itself, meaning there is no need to preset any basis function. However, EMD presents mode mixing, generating oscillations of different and similar scales in a mode or different modes. To curtail this problem, the ensemble empirical mode decomposition (EEMD) was proposed by [17]. The EEMD filters an ensemble of signals with added white noise and treats the mean as the true result. However, the EEMD was not able to sufficiently curtail its limitations. To overcome this problem, we use a variant denoising method of EMD called the complete ensemble empirical mode decomposition with adaptive noise (CEEMDAN) [18] which can achieve an accurate original signal reconstruction. CEEMDAN is a data-driven, non-parametric, and adaptive time-frequency decomposition ensemble model for non-linear and non-stationary signals. It gives an accurate reconstruction of the real signal. Another variant of the EMD called the local mean decomposition technique (LMD) was also proposed to incorporate local means that can better capture the slowly varying components of the signal (data) [19].

Hybrid models using different mode decomposition and machine learning methods have proven effective in forecasting time series data with high nonlinearity, non-stationarity, and uncertainty [20, 21]. These types of models have been applied in different areas, such as rainfall prediction [22], meteorological temperature prediction [15], wind power forecasting [14, 23], finance [24, 25], evapotranspiration forecasting [26], solar irradiance forecasting [27], short-term forecasting of metro passenger flow [28], health [29], agriculture [30]. For instance [22], used ensemble empirical mode decomposition (EEMD) to decompose rainfall data into a set of components, and each component was used as input data for a neural network for forecasting rainfall. [15] used a combined prediction model based on variational mode decomposition-differential symbolic entropy (VMD-DSE) and Volterra to forecast the monthly mean meteorological temperature. [24] predicted the price series of bitcoin using a hybrid of variational mode decomposition (VMD) and a generalized additive model. In their work [28], proposed a forecasting technique for short-term passenger flow using a hybrid of EMD and long-term memory neural networks (LSTM). The results of the constructed EMD-LSTM demonstrate the reliability, feasibility, and robustness of the constructed forecasting model. In a recent study [27], constructed and compared a hybrid model of CNN-LSTM-MLP that is aggregated with variational mode decomposition and error correction to different traditional techniques. The results of the study showed the superiority of the constructed model compared to the traditional models. [31] compared the efficiency of EMD, ensemble EMD (EEMD), and complete ensemble empirical mode decomposition with adaptive noise (CEEMDAN) in decreasing the noise affecting electrocardiogram (ECG) signals. The results showed the robustness of CEEMDAN in decreasing the noise, which obstructed the ECG signals relative to EMD and EEMD. To improve the quality of life for people who have epilepsy and open up new treatment options for human health [29], combined a local mean decomposition algorithm (LMD) and a convolutional neural network (CNN) to predict seizure events using the Freiburg dataset. In the study of [32], the author compared the efficiency of a hybrid robust local mean decomposition (LMD) and artificial neural network (ANN) in predicting streamflow data with different hybrid methods (LMD and support vector machine, LMD and long short-term memory, empirical mode decomposition and ANN, additive autoregressive moving average, and ANN). The results of the study showed that the hybrid LMD-ANN performed better than the other listed hybrid methods.

Clearly, there is extensive literature on single- and two-layer hybrid models that combine decomposition techniques and machine learning methods, as exhibited in the above extensive literature review. However, the single- and two-layer hybrid models are mostly not able to completely predict the non-stationary, non-linear, and often volatile original time series data. Motivated by the above extensive literature review, we propose a novel three-phase hybrid model called the CEEMDAN-LMD-NN prediction model, which integrates CEEMDAN, LMD, and NN for multi-step head temperature prediction. Despite the efficiency of CEEMDAN and LMD in signal decomposition and NN in prediction, a hybrid model using these three techniques has not yet been explored in the temperature prediction literature. The main contributions of this paper can be summarized as follows: (1) A new hybrid model, which involves secondary decomposition, is proposed to conduct temperature multi-step prediction. The applicability of the hybrid model for temperature prediction shows that all three components of this hybrid model are effective and can improve the overall prediction accuracy; (2) verifying the performance of the proposed three-phase CEEMDAN-LMD-NN model by benchmarking the results with similar standalone and two-phase prediction models.

The paper is structured as follows: the theoretical overview explains the theoretical framework; the materials and methodology section presents the data and the proposed prediction model used in the study; the empirical results section provides the analysis of the results of the study; and the conclusion and recommendation of the study are presented in the last section.

## Theoretical overview

In this section, the theoretical frameworks for CEEMDAN, LMD and neural network (NN) are described.

### Complete ensemble empirical mode decomposition with adaptive noise (CEEMDAN)

CEEMDAN gives an exact reconstruction of the original signal and provides a better separation of the IMF with a low computation cost. Define an operator *E*_*k*_(⋅) as the function that, given a signal, produces the *j*_*th*_ modal component obtained by empirical mode decomposition (EMD). Let *ω*^*i*^ be normally distributed white noise, *ω*^*i*^ ∼ *N*(0, 1), *ϵ*_*k*_ be the amplitude of white noise added for the *k*_*th*_ time, and the original signal/targeted data be *X*(*n*). The following steps are used in CEEMDAN decomposition.

*Step 1*: The white noise *X*(*n*) + *ϵ*_0_*ω*^*i*^(*t*) is added to the original signal, and the *I*_*th*_ EMD is performed. *IMF*_1_ is obtained using:

$$\begin{array}{c} IMF_{1}\left(n\right)=\frac{1}{I}\sum_{i=1}^{I}E_{1}(X\left(n\right)+\epsilon_{0}\omega^{i}\left(n\right)) \end{array}$$ (1)

*Step 2*: Compute the first (k = 1) residue component:

$$\begin{array}{c} r_{1}\left(n\right)=X\left(n\right)-IMF_{1} \end{array}$$ (2)

*Step 3*: Decompose *r*_1_(*n*) + *ϵ*_1_*E*_1_(*ω*^*i*^(*n*)), *i* = 1, 2, ⋯, *I* to obtain the first EMD mode. *IMF*_2_ is then computed using the mean value of *IMF*_1_:

$$\begin{array}{c} IMF_{2}\left(n\right)=\frac{1}{I}\sum_{i=1}^{I}E_{1}(r_{1}\left(n\right)+\epsilon_{1}E_{1}\left(\omega^{i}\left(n\right)\right)) \end{array}$$ (3)

*Step 4*: For *k* = 2, 3, ⋯, *K*, the *k*_*th*_ residue is computed as:

$$\begin{array}{c} r_{k}\left(n\right)=r_{k-1}\left(n\right)-IMF_{k}\left(n\right) \end{array}$$ (4)

*Step 5*: Decompose realizations *r*_*k*_(*n*) + *ϵ*_*k*_*E*_*k*_(*ω*^*i*^(*n*)), *i* = 1, 2, ⋯, *I* until their first EMD mode and *IMF*_*k*+1_ can be computed with the mean value of *IMF*_1_ as below:

$$\begin{array}{c} IMF_{k+1}\left(n\right)=\frac{1}{I}\sum_{i=1}^{I}E_{1}(r_{k}\left(n\right)+\epsilon_{k}E_{k}\omega^{i}\left(n\right))) \end{array}$$ (5)

*Step 6*: Repeat Eqs 4 and 5 until the obtained residue is no longer possible to be decomposed, i.e., the residual component does not have no less than two extrema. Finally, the residual component should satisfy:

$$\begin{array}{c} r\left(n\right)=x\left(n\right)-\sum_{k=1}^{K}IMF_{k}, \end{array}$$ (6)

The original data set is decomposed as follows:

$$\begin{array}{c} x\left(n\right)=r\left(n\right)+\sum_{k=1}^{K}IMF_{k} \end{array}$$ (7)

### Local mean decomposition

Local Mean Decomposition (LMD) is a signal processing technique that has a similar principle to empirical mode decomposition (EMD) and is used to decompose a complex signal into simpler components based on their local mean values. LMD was originally developed by [33] in 2005 to process electroencephalogram signals. The LMD approach performs signal decomposition and demodulation by generating the local average and envelope estimation functions, thereby resolving the over-envelope, under-envelope, and modal aliasing problems in the EMD method. Unlike the EMD, the LMD offers the benefits of fewer false components and fewer iterations and may, to some degree, reduce the endpoint effect [34]. As stated by [35], LMD directly calculates instantaneous amplitude (IA) and instantaneous frequency (IF) without using the Hilbert transform (HT), yielding more accurate results.

LMD works by identifying and extracting the different frequency components of a signal through a process of successive filtering and averaging. The basic idea is to decompose a signal into a series of local mean functions and local oscillations. The local mean function represents the slowly varying component of the signal, while the local oscillations represent the high-frequency components. LMD has been extensively applied in the decomposition of electroencephalogram (EEG) signal [29], fault diagnosis for rotating machinery [35] and recently in wind prediction and hydrological time series data [32]. It has been shown to be effective in separating different components of a signal, which can be useful for feature extraction, denoising, and other signal processing tasks.

For a given signal *s*(*t*), the LMD is found by using the moving average method to smooth the signal over time. This averaging takes into account how far apart the signal’s extreme points are. The steps used in implementing the LMD algorithm are as described below [33]:

*Step 1*: Extract all the local extrema *n*_*i*_ from the original signal *s*(*t*). From Eq 8, compute the local mean value *m*_*i*_ of each two successive extrema points,

$$\begin{array}{c} m_{i}=\frac{n_{i+1}+n_{i}}{2} \end{array}$$ (8)

All the local mean values *m*_*i*_ are connected by a straight line that extends between successive extrema points.

*Step 2*: From Eq 9, compute the local envelope estimate function *a*_*i*_ of two successive extrema *n*_*i*_ and *n*_*i*+1_

$$\begin{array}{c} a_{i}=\frac{|n_{i+1}+n_{i}|}{2} \end{array}$$ (9)

*Step 3*: Moving averaging is used to smooth the local means, resulting in a smoothly fluctuating continuous local mean function *m*_11_*t*. To create a continuously variable smooth envelope function *a*_11_*t*, the local magnitudes are smoothed in the same manner and to the same extent as the local means.

*Step 4*: Compute the residue signal *h*_11_*t* (see Eq 10) by subtracting the local mean function *m*_11_*t* from the original signal *s*(*t*),

$$\begin{array}{c} h_{11}t=s\left(t\right)-m_{11}t \end{array}$$ (10)

A frequency modulated signal *s*_11_*t* is then calculated using the computed *h*_11_*t* and *a*_11_*t* as

$$\begin{array}{c} s_{11}t=\frac{h_{11}t}{a_{11}t} \end{array}$$ (11)

*Step 5*: The envelope estimate *a*_12_*t* of *s*_11_*t* are obtained by repeating Steps 1 to 3. For *a*_12_*t* = 1, the inner iteration is stopped and *s*_12_*t* is taken as the first purely frequency modulated (FM) signal. Otherwise, the *s*_11_*t* is taken as the original signal, and Steps 1-4 are repeated *n* times until the condition *a*_1(*n*+1)(*t*)_ of a purely frequency modulated signal *s*_1*n*_(*t*) satisfies *a*1(*n* + 1)(*t*) = 1. Hence, the initial iteration can be described as follows:

$$\begin{array}{c} \left\{ \begin{array}{c} h_{11}\left(t\right)=s\left(t\right)-m_{11}t\\ h_{12}\left(t\right)=s_{11}\left(t\right)-m_{12}t\\ \cdots \\ h_{1n}\left(t\right)=s_{1(n-1)}\left(t\right)-m_{1n}t \end{array}\right. \end{array}$$ (12)

where

$$\begin{array}{c} \left\{ \begin{array}{c} s_{11}\left(t\right)=\frac{h_{11}t}{a_{11}t}\\ s_{12}\left(t\right)=\frac{h_{12}t}{a_{12}t}\\ \cdots \\ s_{1n}\left(t\right)=\frac{h_{1n}t}{a_{1n}t} \end{array}\right. \end{array}$$ (13)

*Step 6*: Compute the corresponding instantaneous amplitude of the first product function (*PF*_1_) by multiplying all the smoothed local envelopes from the iterations,

$$\begin{array}{c} a_{1}\left(t\right)=a_{11}\left(t\right)a_{12}\left(t\right)\cdots a_{1n}\left(t\right)=\prod_{p=1}^{n}a_{1p}t \end{array}$$ (14)

where

$$\begin{array}{c} \underset{n\to \infty }{\text{lim}}\;a_{1n}\left(t\right)=1 \end{array}$$ (15)

*Step 7*: Construct *PF*_1_ from *a*_1_(*t*) and *s*_1*n*_*t* as follows:

$$\begin{array}{c} PF_{1}\left(t\right)=a_{1}\left(t\right)s_{1n}\left(t\right) \end{array}$$ (16)

Theoretically, majority of the oscillation information of *s*(*t*) is embedded in *PF*_1_(*t*). The instantaneous amplitude of *PF*_1_(*t*) and the instantaneous frequency can be computed as

$$\begin{array}{c} f_{1}\left(t\right)=\frac{1}{2\pi }\frac{d[\text{arccos}\left(s_{1n}\left(t\right)\right)}{dt} \end{array}$$ (17)

*Step 8*: Calculate the residue signal *u*_1_(*t*) which is regarded as the new signal. The above steps are repeated *n* times while *u*_*n*_(*t*) is free of any oscillation. This process can be given as,

$$\begin{array}{c} \left\{ \begin{array}{c} u_{1}\left(t\right)=s\left(t\right)-PF_{1}\left(t\right)\\ \;\;\vdots \\ u_{n}\left(t\right)=u_{n-1}\left(t\right)-PF_{n}\left(t\right) \end{array}\right. \end{array}$$ (18)

Finally, the original signal *s*(*n*) can be given as the sum of the PFs and residue signal,

$$\begin{array}{c} s\left(t\right)=\sum_{q=1}^{n}PF_{n}\left(t\right)+u_{n}\left(t\right) \end{array}$$ (19)

where *n* is the number of Pfs.

A summarized algorithm of the LMD method is presented in Algorithm 1.

**Algorithm 1**: Local Mean Decomposition

**Input**: Signal *s*(*t*), number of decomposition levels *L*

**Output**: Envelopes *e*_*l*_(*t*) and oscillatory components *h*_*l*_(*t*) for *l* = 1, …, *L*

**for** *l* = 1 **to** *L* **do**

 Compute the envelope of *s*(*t*) using a moving average filter:

 *e*_*l*_(*t*) ← MovingAverage(*s*(*t*)); Compute the oscillatory component:

 *h*_*l*_(*t*) ← *s*(*t*) − *e*_*l*_(*t*); Update the signal for the next iteration: *s*(*t*) ← *h*_*l*_(*t*);

**end**

### Artificial neural network

Artificial neural networks (NN), also known as neural networks, are a class of machine learning algorithms that are inspired by the structure and operation of the human brain. Neurons, the interconnected nodes that make up layers in neural networks, process input and generate predictions based on that information. Each neuron takes in one or more inputs (*s*_1_, *s*_2_, …*s*_*p*_), and multiply each by a weight (*w*_1_, *w*_2_, …*w*_*p*_), and adds them together $\sum_{i=1}^{N}(w_{i}s_{i})$. The result passes through an activation function *g*[(*w*_*i*_*s*_*i*_)], which determines whether the neuron “fires” or not. A constant *h* termed the bias, is added to the sum of the multiplied weight and input to enable the entire activation function to be moved to the left or right to produce the necessary output values. This gives a new function as *g*[(*w*_*i*_*s*_*i*_ + *h*)]. Nonlinear activation functions are always considered in neural networks because of the nonlinear mapping required in the system. Such nonlinear activation functions include the hyperbolic tangent function and the sigmoid function. The neuron’s output is then sent to the next layer of the neural network, which usually includes an input layer, one or more hidden layers, and an output layer. The raw data, such as a value, image, or sentence, is fed into the input layer, and the output layer creates a prediction or classification based on that data. The hidden layers are where most of the computation takes place, and where the neural network learns to recognize patterns and make predictions. The neural network’s learning rate is built on the two techniques of optimization and backpropagation.

**Fig 1:**
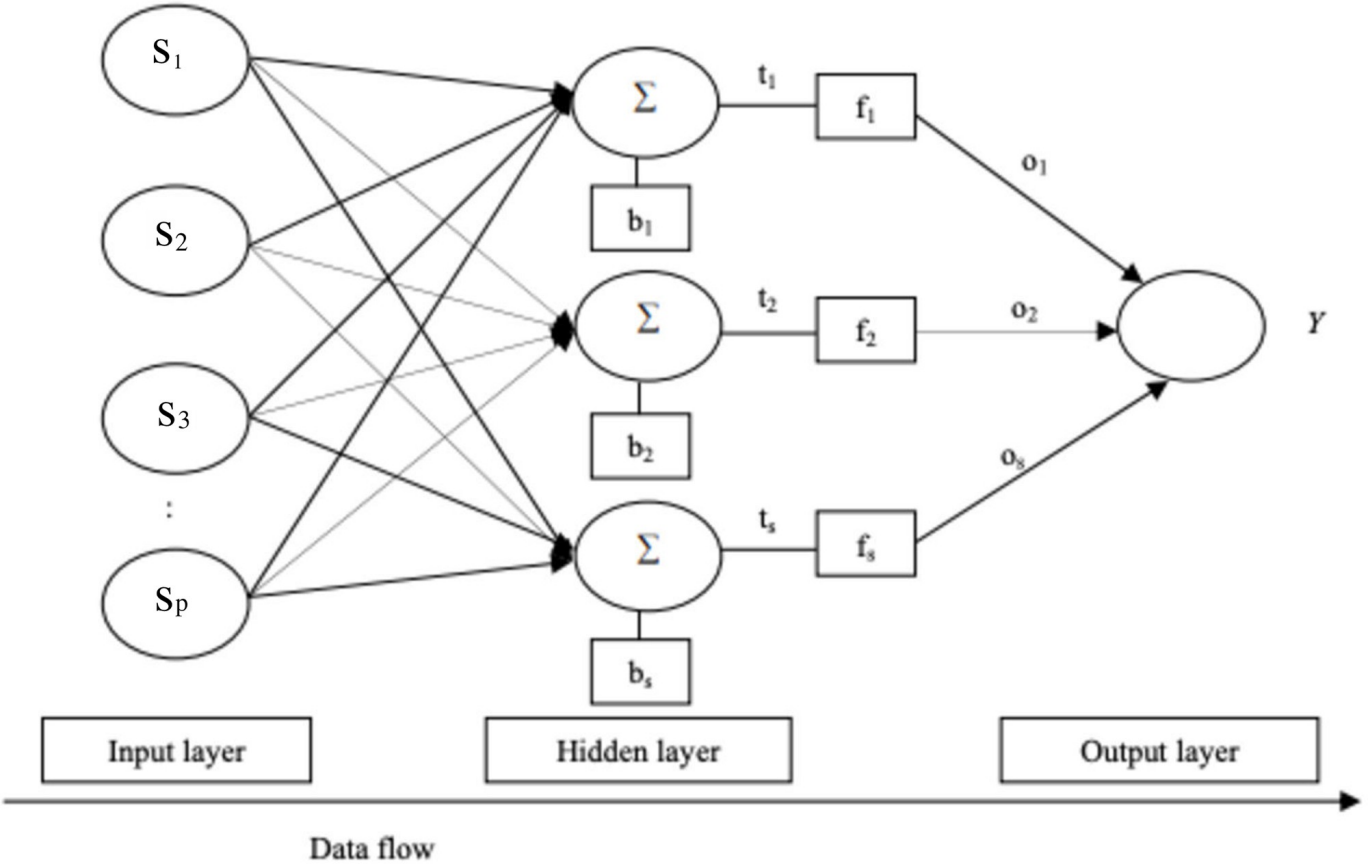
ANN architecture used in this study.

Backward propagation of errors, also known as backpropagation, is the algorithm used in calculating the gradient of the loss function regarding its weights and bias. The backpropagation considers the mean square error (MSE), mostly called the cost function, represented as $\frac{1}{n}\sum_{i=1}^{N}(O_{i}-\bar{O_{i}}{)}^{2}$, where *O*_*i*_ is the actual output and $\bar{O_{i}}$ represents the targeted output. In order to find the optimal weights and bias for our perceptron, we must first understand how the cost function changes in relation to weights and bias. This can be accomplished through the use of gradients (rates of change) generated by a partial differential equation. Fig 1 shows the network architecture of a feed-forward neural network used in this study.

## Materials and methodology

The data and methodology used for constructing the proposed prediction model (CEEMDAN-NN) and the evaluation metrics used are discussed.

### Study area

To verify the validity of the proposed three-phase CEEMDAN-LMD-NN model, the Upper East region of Ghana was selected. The Upper East region is located in the northern part of Ghana, occupying 2.7% of the total land area in Ghana. The Upper East region lies between longitudes of 0° and 1° West and latitudes of 10° 30’N and 11°N. Northern Ghana (including the Upper East Region) experiences temperature extremes with a wide daily and annual range of temperatures. The daytime temperatures at normal periods for both regions range from 28°*C* to 40°*C*. An abrupt increase in temperature is experienced in March, April, and May when the average temperature exceeds 30°*C*. Major crops grown in the Upper East region include maize, millet, and sorghum as cereals; legumes include bambara beans, groundnut, and cowpea; and roots and tubers are yam and cassava. Its temperature data is reliable because of the availability of a weather station at these locations.

### Temperature datasets

This paper employs the monthly temperature data of the Upper East region of Ghana as a representative of the temperature data to test the prediction efficiency of the three-phase CEEMDAN-LMD-NN model constructed. The temperature data was obtained from the Climate Change Knowledge Portal (CCKP) Climatic Research Unit Timeseries (CRU TS). The CCKP data set was chosen because of its availability to the public and also because it compiles station data for multiple variables from numerous data sources into a consistent format. The CCKP data set is gridded to 0.5° × 0.5° resolution, based on analysis of over 4000 individual weather station records. For the purpose of this study, the sample data covers a period of 384 months from January 1990 to December 2021 in the Upper East Region of Ghana.

**Fig 2:**
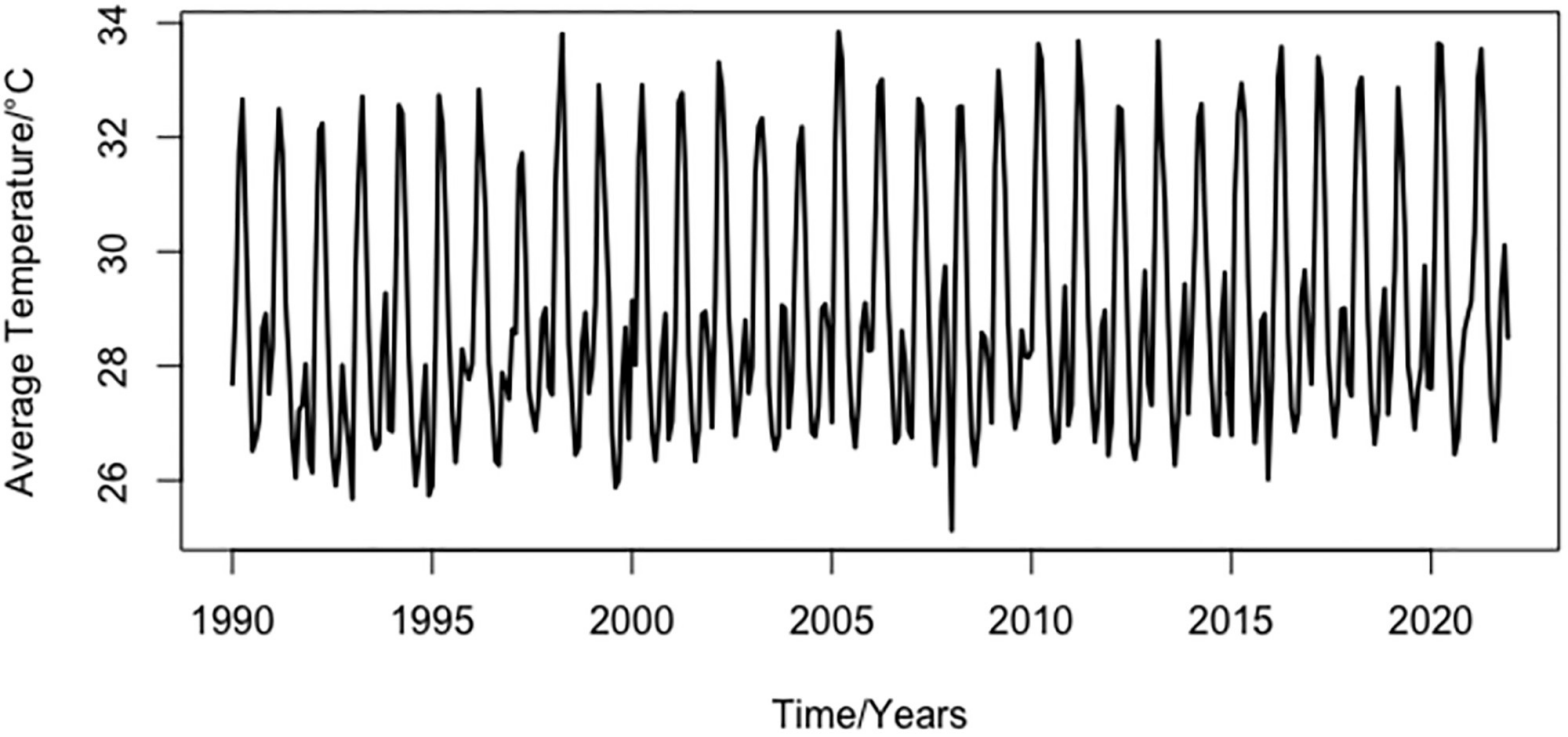
Average monthly temperature of Upper East from 1990 to 2021.

Fig 2 shows the plot of the mean temperature for the Upper East from January 1990 to December 2021. The descriptive statistics are presented in Table 1. The maximum (33.84°*C*) and minimum (25.13°*C*) average temperature values were recorded in March 2005 and January 2008, respectively. This is consistent with the previous statement that average temperatures increase from March to May each year. Clearly, from Table 1, the skewness and kurtosis values are significant at the 1% level (p-value less than 1% is considered statistically significant) and deviate from the value of a normal distribution. However, the deviation of the data from the mean value is small (2.16°*C*). This indicates that the temperature in this region is not normally distributed.

**Table 1:** Descriptive statistics of the mean monthly temperature of Upper East and Upper West regions, Ghana.

Subnational Unit	Count	Min (°*C*)	Max (°*C*)	Mean (°*C*)	Std. Dev.	Skewness	Kurtosis
Upper East	384	25.13	33.84	28.98	2.16	0.6358***	-0.7629***

*** represents the significance level at 1%

### Construction of the three-phase hybrid CEEMDAN-LMD-NN prediction model

**Fig 3:**
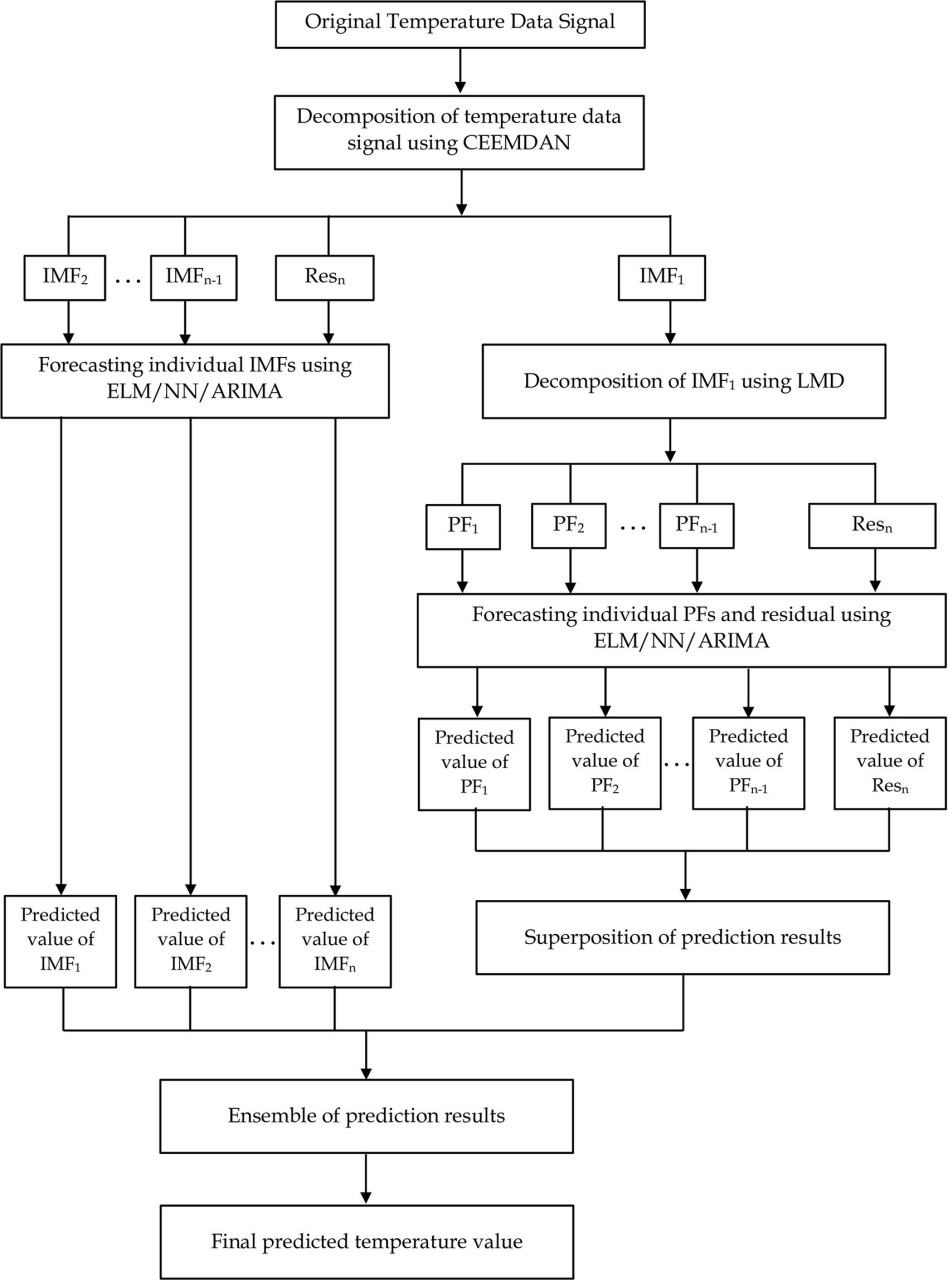
Flowchart of the three-phase hybrid CEEMDAN-LMD-NN model integrating complete ensemble empirical mode decomposition adaptive noise (CEEMDAN), local mean decomposition (LMD), and neural network (NN) algorithm.

The flowchart of the three-phase hybrid CEEMDAN-LMD-NN model is illustrated in Fig 3.

In the first phase, the CEEMDAN algorithm is employed to decompose the original temperature signal series into distinct intrinsic mode functions (IMFs) and residue (Res) components. In the second stage, permutation entropy (PE) is used to detect the IMFs and residue with the highest frequency oscillatory behaviour (in our case, IMF_1_ was selected as the IMF with the highest frequency because it had the highest PE). This is necessary because such an IMF or residue (IMF_1_) might contain the unresolved physical structure of the original temperature signal, which can undoubtedly complicate the prediction procedure and lead to less-than-satisfactory results; hence, a second-stage decomposition process is performed. The local mean decomposition (LMD) algorithm is employed to split the high frequency IMF_1_ into different product functions (PFs) and residue. A neural network (NN) is then applied to predict the remaining IMFs and residue sub-series from the CEEMDAN decomposition. The predicted high-frequency IMF_1_ is obtained by aggregating the predicted NN results from the individual PFs and residue components from the local mean decomposition. In the third-phase, all the prediction results are combined to get a final predicted temperature signal. To benchmark the three-phase CEEMDAN-LMD-NN model, predictions are also made using a two-phase CEEMDAN-NN model (without a second decomposition of IMF_1_) and a standalone model without temperature decomposition (that is, only a standalone NN model).

The following subsection gives details of the steps used in constructing the CEEMDAN-LMD-NN prediction model and the analysis therein.

#### Step 1: Data decomposition using CEEMDAN and feature engineering

**Fig 4:**
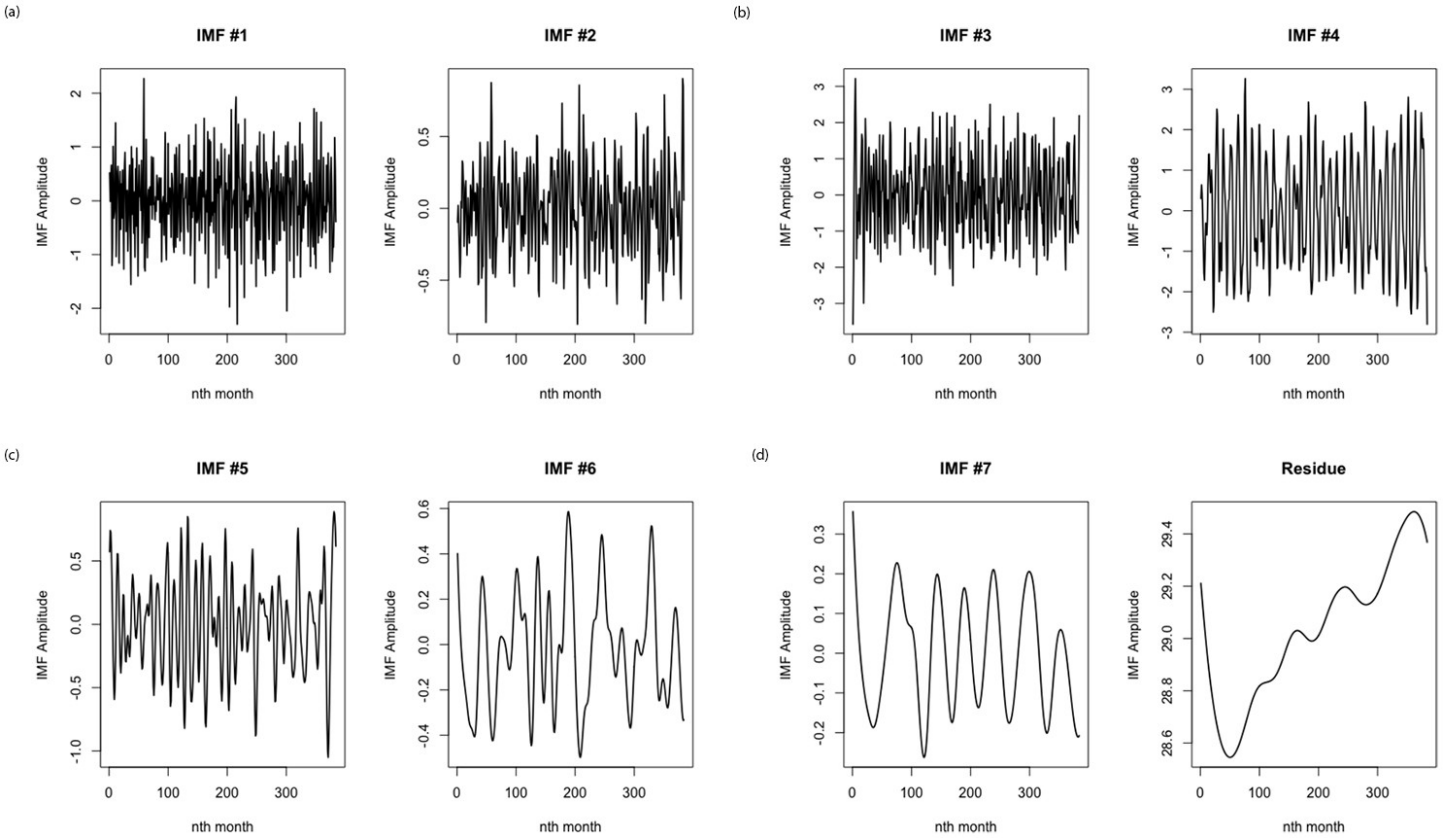
Decomposition of original signal using CEEMDAN to IMFs.

The original temperature data *x* is decomposed into distinct IMFs (sub-series) using the CEEMDAN algorithm, where *IMF*_1_, *IMF*_2_, ⋯, *IMF*_*n*_ − 1, *Res*_*n*_ for *n* = 1, 2, ⋯, *K* represent the obtained intrinsic mode function (IMF) and the residue (Res). For the CEEMDAN algorithm, the standard deviation of the Gaussian random numbers used as additional noise and the number of copies of the input signal used as the ensemble are 2 and 50 respectively. The noisy mean temperature is decomposed into seven (7) IMFs and a residue (see Fig 4). From Fig 4, the behaviour of each of the IMFs is distinct. Clearly, from the figure, IMF 3 and 4 have the largest errors. This is consistent with the standard deviation values obtained from the descriptive statistics of the IMFs and the residue presented in Table 2. From Table 2, all the IMFs are significant at the 1% significance level except the residue (Res). This is desirable because the use of non-stationary time series data in models produces unreliable and spurious results and normally leads to poor understanding and prediction.

**Table 2:** Descriptive statistics of the mean monthly temperature of Upper East and Upper West regions, Ghana.

Component	Mean	Std. Dev.	Skewness	Kurtosis	ADF Test
IMF1	0.0033	0.7407	-0.0912	0.0030	-6.9775***
IMF2	-0.0072	0.3065	0.0873	-0.0489	-9.3403***
IMF3	-0.0039	1.1088	0.0262	-0.4354	-12.936***
IMF4	-0.0211	1.2999	0.1353	-0.9138	-12.268***
IMF5	0.0009	0.3708	-0.07497	-0.2532	-7.6337***
IMF6	-0.0223	0.2479	0.2949	-0.5396	-5.7255***
IMF7	-0.0012	0.1383	0.0746	-1.1582	-4.7724***
Res	29.0314	0.2626	-0.1185	-0.7981	-0.5359

*** represents the significance level at 1%.

By employing the sample entropy (SE) algorithm as in [36], the IMF, or residue with the largest frequency, is identified. The value of sample entropy increases as the complexity of time series increases, and vice versa. From Table 3, it is clear that IMF1 has the highest SE value; hence, it is selected as the IMF that exhibited the maximum frequency. This is essential due to the fact that such an IMF (IMF_1_) could retain the unresolved physical structure of the original temperature signal, which can surely make the prediction technique more difficult and lead to outcomes that are less than desirable.

**Table 3:** Sample entropy of each CEEMDAN IMF and residue signal.

IMF	IMF1	IMF2	IMF3	IMF4	IMF5	IMF6	IMF7	Residue
Sample Entropy	0.8512	0.7661	0.8175	0.5972	0.4806	0.3101	0.2032	0.1517

#### Step 2: Data normalization and splitting

The min-max normalization technique is used to normalize the data points in the range (0, 1). This technique is employed because it preserves the relationships among the original data values. The normalized data is split into two groups (training and testing data). The first 307 data points (80% of the total data) of the original data and IMFs are used as the training data, and the remaining 77 data points are used for testing the constructed prediction models.

**Fig 5:**
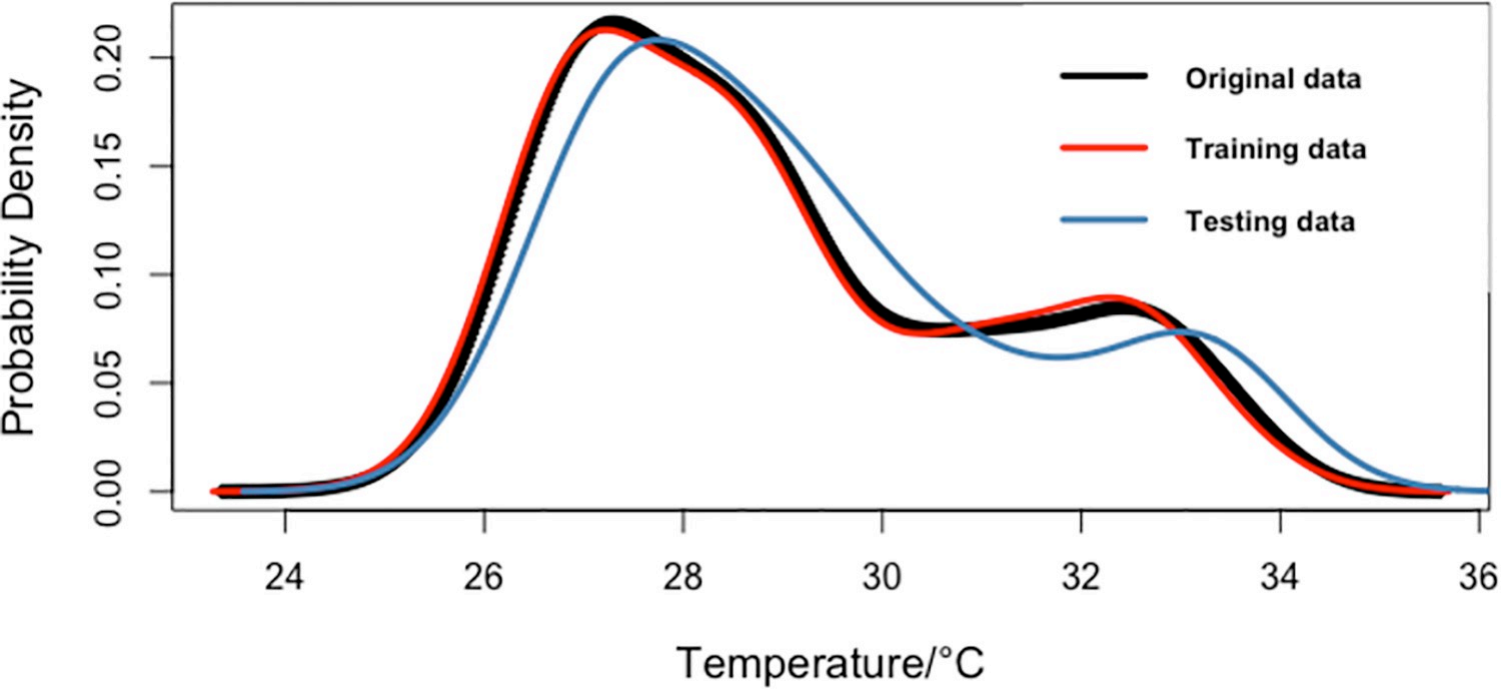
Density of the mean temperature for the original total period, training data, and testing data.

To verify whether the training and testing data are a good representation of the original data, this study employed the kernel density estimation (KDE) curve. The KDE curve is a nonparametric approach used to estimate the unknown density function in probability theory, and it is used to obtain the distribution of the original data, training data, and testing data. The sum of the areas under the KDE curve equals 1. Clearly, from Fig 5, the distribution of the training and testing data periods is similar to the distribution of the original data, which is a requirement for machine learning algorithms. Hence, the training and testing data set is a good representation of the total period under study. From the descriptive statistics in Table 4, it can be verified that the mean, standard deviation, and skewness are homogeneous. This further confirms that the training and testing data are a good representation of the original data.

**Table 4:** Summary statistics of the total training and testing temperature data.

Statistics	N	Mean	Maximum	Minimum	Standard Deviation	Skewness
Total	384	25.13	33.84	28.98	2.15	0.64
Training	307	25.13	33.84	28.92	2.16	0.62
Testing	77	26.02	33.64	29.21	2.15	0.72

#### Step 3: Local mean decomposition of IMF1

**Fig 6:**
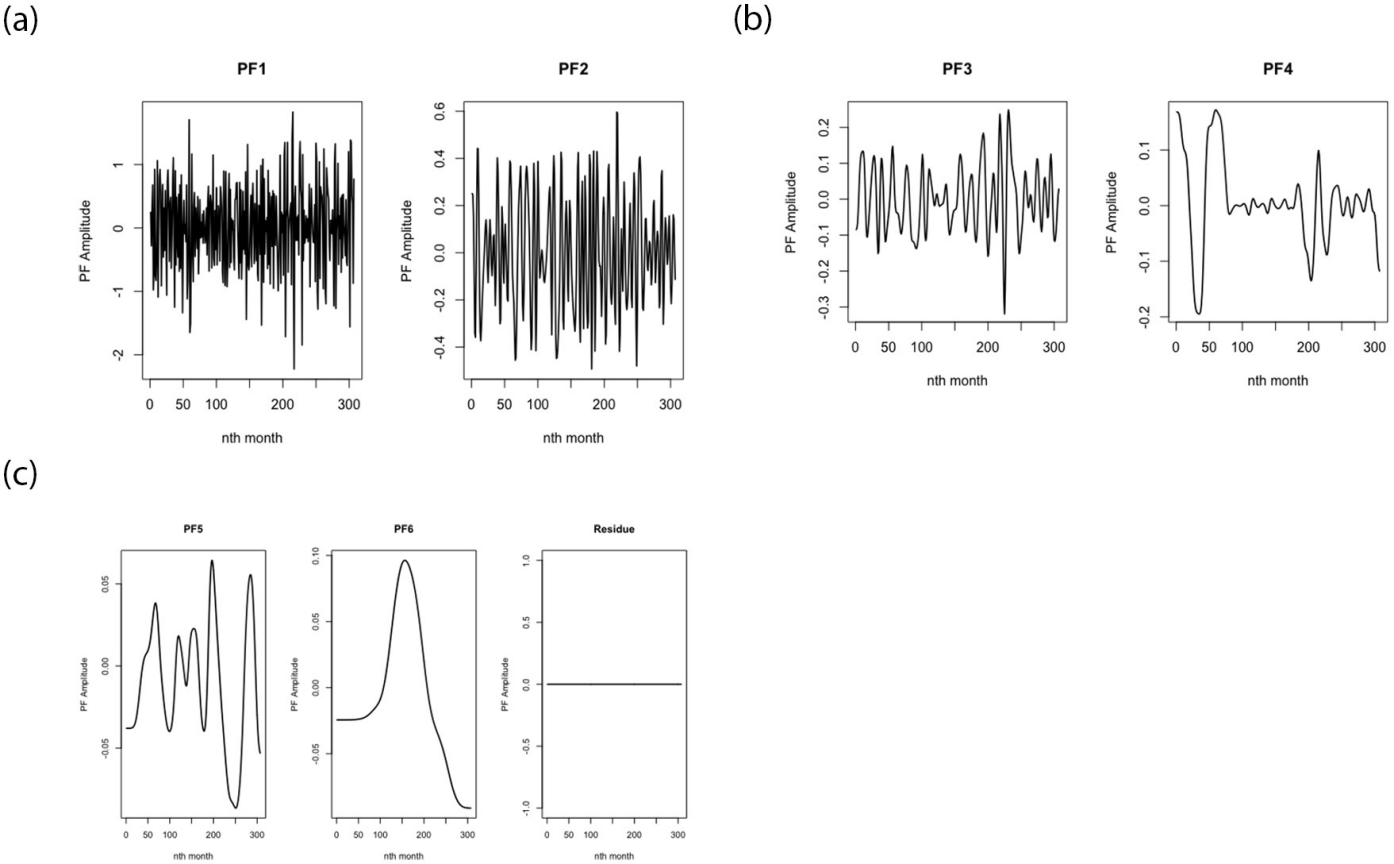
Decomposition of the IMF1 using LMD to PFs.

The local mean decomposition (LMD) algorithm is employed to split the high frequency IMF1 into different product functions (PFs) and residue. The result from the decomposition is as presented in Fig 6. Six product functions (PFs) and a residue (*u*_*n*_(*t*)) are generated from LMD. It is evident from Fig 6 that the PF amplitude is zero for all the months. This indicates that the local mean and local oscillations of the signal are well-behaved and can be effectively decomposed and analyzed using LMD. Each of the product functions (PF1-PF6) and the residue are used as inputs for the feed-forward artificial neural network for prediction.

#### Step 4: Optimization of hyper-parameter and model implementation

By fine-tuning, the most effective hyperparameter values of the ANN and ELM are selected based on the training data. In sequel to that, we evaluate the prediction accuracy of the proposed CEEMDAN-LMD-NN model and other benchmarking models to prove the efficiency of the proposed model.

*ARIMA optimal model selection*. For optimal selection of the parameter values of the ARIMA model, this study uses the “auto.arima” function in the “forecast” package of the R statistical software. The function returns the best ARIMA model according to either the Akaike Information Criterion (AIC) or the Bayesian Information Criterion (BIC) values generated. The lower the value of either the AIC or BIC, the more optimal the model.

*ELM model tuning*. For all the steps-ahead prediction and for the standalone, two-phase, and three-phase models, the ELM model employed a “lasso” estimation. The number of hidden neurons (hd) for all the phase and step-ahead predictions was set within the range of 20 to 200. The univariate lags (L) to use as inputs were set as 1: *frequency*(*inputsignal*). The number of networks to train (reps) is also set in the range of 10 to 50 repetitions.

*ANN model tuning*. This study uses feed-forward neural networks with a single hidden layer and lagged inputs for predicting univariate time series. The size of the model (number of nodes in the hidden layer) ranged from 2 to 8; repeats (the number of networks to fit with different random starting weights) ranged from 1 to 8; and maximum iteration (maxit) ranged from 50 to 200.

### Model evaluation

The prediction performance of the constructed three-phase hybrid CEEMDAN-LMD-NN is assessed using different evaluation metrics: root mean square error (RMSE), mean absolute error (MAE), mean absolute percentage error (MAPE), percentage bias (P-Bias), and the ratio of RMSE to standard deviation (STD) of the observations (RSR). As widely used evaluation metrics, the RMSE, MAE, and MAPE give prediction ability where a small value is achieved by a perfect model. When the error distribution is expected to be Gaussian, RMSE deduces the global fitness and is more appropriate to represent model performance than MAE, whereas MAE provides a more balanced perspective of the goodness-of-fit based on estimation errors [37]. While P-Bias evaluates the average tendency of predicted values to be bigger or smaller than observed values, RSR measures residual variation that differs from zero (indicating zero RMSE or residual variation). P-Bias has an optimal value of 0, with low-magnitude values indicating accurate predictions, positive values indicating overestimation bias, and negative values indicating model underestimation bias. The cross-validation of the observed and predicted temperatures is also performed using diagnostic plots such as the error histogram, scatter diagrams, and Taylor plots [38].

$$\begin{array}{c} RMSE=\sqrt{\frac{1}{n}\sum_{d=1}^{n}{\left(T_{d}^{p}-T_{d}^{o}\right)}^{2}} \end{array}$$ (20)

$$\begin{array}{c} MAE=\frac{1}{n}\sum_{d=1}^{n}|T_{d}^{p}-T_{d}^{o}| \end{array}$$ (21)

$$\begin{array}{c} MAPE=\frac{1}{n}\sum_{d=1}^{n}\frac{\left|T_{d}^{o}-T_{d}^{p}\right|}{T_{d}^{o}} \end{array}$$ (22)

$$\begin{array}{c} RSR=\frac{\text{RMSE}}{\text{STD}}=\frac{\sqrt{\sum_{d=1}^{n}{\left(T_{d}^{p}-T_{d}^{o}\right)}^{2}}}{\sqrt{\sum_{d=1}^{n}{\left(T_{d}^{o}-{\bar{T}}^{o}\right)}^{2}}} \end{array}$$ (23)

$$\begin{array}{c} \text{P-Bias}=\frac{\sum_{d=1}^{n}\left(T_{d}^{o}-T_{d}^{p}\right)}{\sum_{d=1}^{n}T_{d}^{o}}\times 100 \end{array}$$ (24)

where: *n* is the number of input observations; $T_{d}^{p}$ and $T_{d}^{o}$ are the predicted and observed temperatures at day *d*; ${\bar{T}}^{p}$ and ${\bar{T}}^{o}$ are the averages of the predicted and observed temperatures.

For a perfect prediction of the temperature data at the observed study area, an RMSE/MAE/MAPE/RSR/P-Bias ≈0 are desired. According to literature, a model is very good if the RSR is less than 0.50, good if it is greater than 0.65 and less than 0.6, and satisfactory if it is greater than 0.5 and less than 0.70 [39].

## Empirical results

### Analysis of single-phase prediction models

In Table 5, an artificial neural network (NN) model is benchmarked against an extreme learning machine (ELM) and aurogressive integrated moving average (ARIMA) models for prediction 2-, 4-, and 6-month-ahead temperatures at the Upper East station. Considering the 2-month (short-term) prediction horizon, the values from the model evaluation metrics shows that the ARIMA model outperformed the NN and ELM models. However, the NN model outperformed the ARIMA and ELM prediction models when considering the 4-month (medium-term) and 6-month (long-term) prediction horizons. Although the accuracy of the 4- and 6-month-ahead predicted temperature values is significantly lower than the 2-month ahead prediction, it is clear from these results that the NN model consistently outperforms the ARIMA and ELM models when it comes to temperature prediction at longer lead time horizons.

**Table 5:** The 2-month, 4-month, 6-month ahead prediction performance comparison of the temperature data for different standalone prediction models.

Model	Upper East				
	RMSE (°*C*)	MAPE (%)	MAE (°*C*)	P-Bias(%)	RSR
*2-month ahead*					
ARIMA	**0.3746**	**0.0134**	**0.3634**	**0.0032**	**0.7461**
ELM	0.9994	0.0369	0.9977	-0.0369	1.9907
NN	0.4603	0.0167	0.4523	0.0167	0.9168
*4-month ahead*					
ARIMA	1.0872	0.0318	0.9061	0.0267	0.9888
ELM	0.7121	0.0205	0.5565	-0.0205	0.6477
NN	**0.6487**	**0.0183**	**0.5122**	**0.0179**	**0.5900**
*6-month ahead*					
ARIMA	1.7151	0.0527	1.4455	-0.0137	1.4981
ELM	1.4289	0.0399	1.0664	-0.0399	1.2481
NN	**0.8708**	**0.0271**	**0.7381**	**-0.0029**	**0.7606**

### Analysis of two-phase models

A closer comparison of the performance metrics for different lead times (see Table 6) shows that the results for the longest lead time (i.e., 6 months) are in fact quite good for the two-phase hybrid CEEMDAN-NN model in terms of all the performance metrics used. The P-Bias of the hybrid CEEMDAN-NN model is relatively small, which concurs with the relatively small RMSE, MAPE, MAE, and RSR values. However, for the 2- and 4-month ahead prediction, the two-phase CEEMDAN-ARIMA performed relatively better than the CEEMDAN-ELM and CEEMDAN-NN. This is evident from the small values recorded for the RMSE, MAPE, MAE, P-Bias, and the RSR.

**Table 6:** The 2-step, 4-step, 6-step-ahead prediction performance comparison of the temperature data for different two-phase prediction models.

Model	Upper East				
	RMSE (°*C*)	MAPE (%)	MAE (°*C*)	P-Bias(%)	RSR
*2-month ahead*					
CEEMDAN-ARIMA	**0.3995**	**0.0157**	**0.4512**	**-0.0057**	**0.7974**
CEEMDAN-ELM	1.0234	0.0369	0.9921	-0.0369	2.0385
CEEMDAN-NN	0.6143	0.0226	0.6092	-0.0226	1.2236
*4-month ahead*					
CEEMDAN-ARIMA	**0.3923**	**0.0121**	**0.4668**	**-0.0023**	**0.3749**
CEEMDAN-ELM	0.7287	0.0205	0.5556	-0.0205	0.6627
CEEMDAN-NN	0.4701	0.0148	0.4059	-0.0140	0.4276
*6-month ahead*					
CEEMDAN-ARIMA	1.3790	0.0323	0.8606	-0.0298	1.2045
CEEMDAN-ELM	1.5379	0.0432	1.1549	-0.0432	1.3433
CEEMDAN-NN	**1.2172**	**0.0292**	**0.7764**	**-0.0261**	**1.0632**

### Analysis of three-phase models

**Fig 7:**
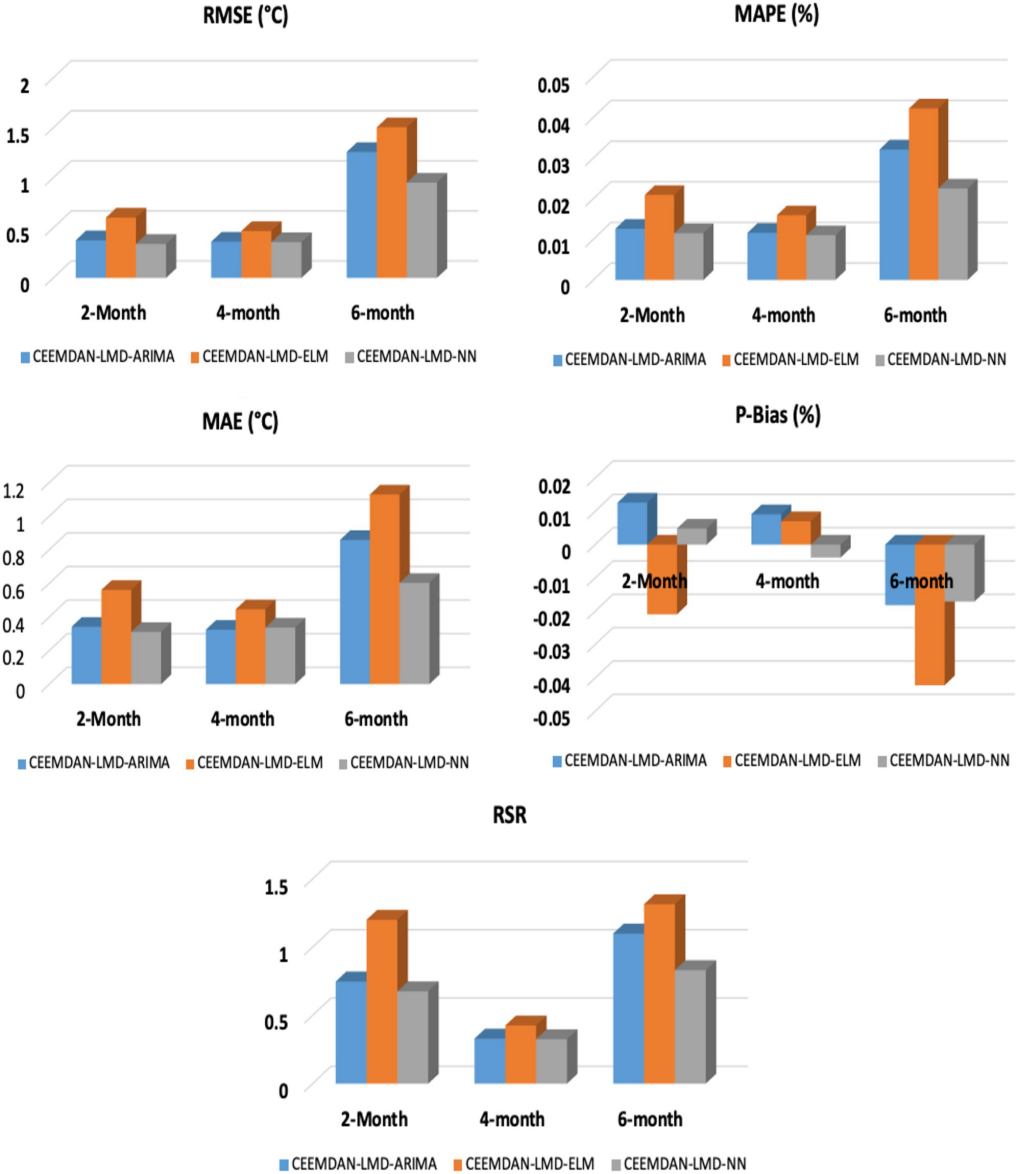
A comparison of RMSE, MAPE, MAE, P-Bias, and RSR for the step-ahead forecasts generated by the different hybrid three phase prediction models.

**Fig 8:**
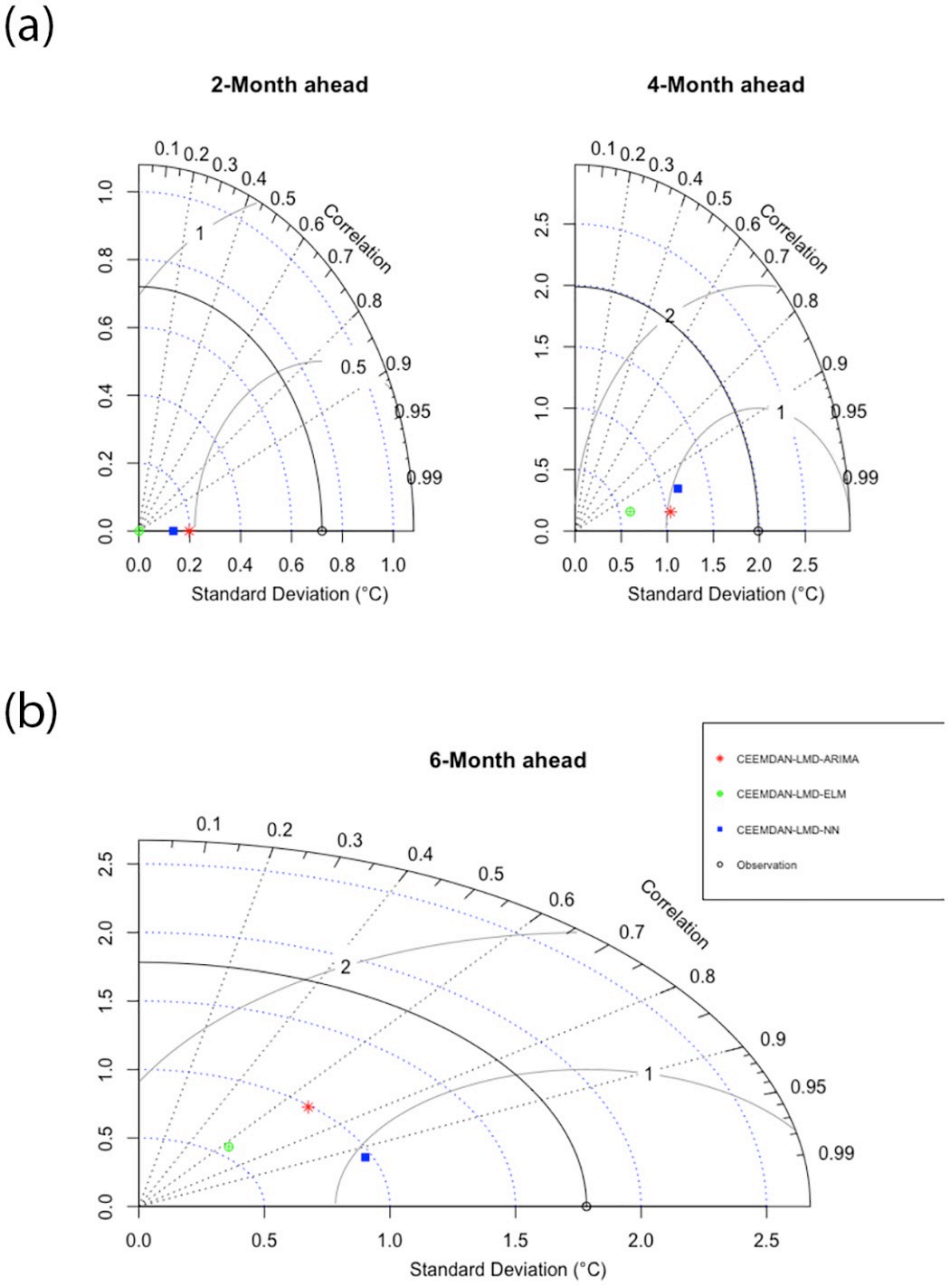
Taylor plot for CEEMDAN-LMD-ARIMA, CEEMDAN-LMD-ELM, and CEEMDAN-LMD-NN.

Table 7 shows the performance of the 2-step, 4-step, and 6-step-ahead for the different three-phase prediction models. For all the lead times, the three-phase CEEMDAN-LMD-NN had optimal performance as compared to the CEEMDAN-LMD-ELM and CEEMDAN-LMD-ARIMA prediction models. Even though the two-phase CEEMDAN-ARIMA model performed optimally when compared to the CEEMDAM-NN, it is evident from Table 7 that the three-phase CEEMDAN-LMD-NN had the lowest prediction errors for both the short-term, medium-term, and long-term prediction. This shows that hybridizing LMD with CEEMDAN and NN leads to an optimal prediction of temperature values for short-term, medium-term, and long-term prediction temperature prediction. Even though, the statistical metrics have been able to evaluate the performance of the three-phase models, error plots and Taylor diagrams are also important when assessing the temporal correspondence of observed and predicted values. Fig 7 shows the comparison of the different error plots (RMSE, MAPE, MAE, P-Bias, and RSR) for the 2-, 4-, and 6-step-ahead forecasts generated by the different hybrid three phase prediction models. From the figure, it is evident that the error values for the long-term prediction (6 months ahead) are high as compared to the short-term (2 months) and medium-term (4-months) ahead prediction. The relative merits of the different models for the 2-month, 4-month, and 6-month ahead prediction models can be deduced from Fig 8. For each prediction model, three statistics are plotted: the standard deviation of the forecasted model, the Pearson correlation coefficient, and the centered root mean square (RMS) error. From the 2-month-ahead Taylor plot, it is evident that the correlation coefficient for all the three-phase prediction models to the observation is 1. With regards to the 6-month ahead Taylor plot, the correlation coefficient and the standard deviation of the CEEMDAN-LMD-NN prediction model are about 0.99 and 0.8°*C*. However, the standard deviation of the CEEMDAN-LMD-ELM for the 6-month ahead prediction model is the lowest (about 0.6°*C*). Clearly, the standard deviation for all the prediction models for the 2-month, 4-month, and 6-month aheads is smaller than the standard deviation of the observed.

**Table 7:** The 2-step, 4-step, 6-step-ahead prediction performance comparison of the temperature data for different three-phase prediction models.

Model	Upper East				
	RMSE (°*C*)	MAPE (%)	MAE (°*C*)	P-Bias(%)	RSR
*2-month ahead*					
CEEMDAN-LMD-ARIMA	0.3757	0.0126	0.3414	0.0126	0.7483
CEEMDAN-LMD-ELM	0.6026	0.0209	0.5611	-0.0209	1.2003
CEEMDAN-LMD-NN	**0.3394**	**0.0115**	**0.3111**	**0.0048**	**0.6760**
*4-month ahead*					
CEEMDAN-LMD-ARIMA	0.3622	0.0116	**0.3242**	0.0091	0.3294
CEEMDAN-LMD-ELM	0.4682	0.0159	0.4453	0.0070	0.4258
CEEMDAN-LMD-NN	**0.3579**	**0.0110**	0.3371	**-0.0039**	**0.3255**
*6-month ahead*					
CEEMDAN-LMD-ARIMA	1.2586	0.0321	0.8586	-0.0182	1.0994
CEEMDAN-LMD-ELM	1.5053	0.0422	1.1287	-0.0422	1.3148
CEEMDAN-LMD-NN	**0.9521**	**0.0225**	**0.6027**	**-0.0171**	**0.8316**

### Benchmarking results from the single-, two-, and three-phase prediction models

The optimal three-phase hybrid model (CEEMDAN-LMD-NN) is benchmarked against the optimal single- and two-phase models of the 2-, 4-, and 6-month-ahead temperature prediction. For the 2-month ahead prediction, the CEEMDAN-LMD-NN model yielded the smallest RMSE value of 0.3394°*C* as compared to an RMSE values of 0.3746°*C* and 0.3995°*C* for ARIMA and CEEMDAM-ARIMA respectively. This result is consistent with the relatively low values of the MAPE, MAE, and RSR. However, the ARIMA model recorded the lowest percentage bias (0.0032%). These findings suggest that the CEEMDAN-LMD-NN model may produce reliable and accurate 2-month-ahead predictions.

Similar to the high performance of the three-phase CEEMDAN-LMD-NN model for the 2-month ahead prediction, the performance of the three-phase CEEMDAN-LMD-NN was optimal when benchmarked with the single-phase NN and the two-phase CEEMDAN-NN prediction models for the 4-month ahead prediction. With very low RSR values (0.3255) which are assumed to be an indicator of good performance [39], it can be concluded that the three-phase CEEMDAN-LMD-NN hybrid prediction model can produce acceptable short- to long-term temperature predictions.

A detailed comparison of the performance metrics for the long-term (6-months) prediction results show that the three-phase hybrid CEEMDAN-LMD-NN model is in fact optimal in terms of a relatively small MAPE and MAE. In contrast, the RMSE and RSR values of the single-phase NN model are significantly lower than the hybrid models, indicating a similar efficiency of the single-phase model in predicting long-term temperature values. In fact, the analysis shows that the single-phase NN model has a significantly lower percentage bias error (P-Bias = -0.0029%) compared to the CEEMDAN-ELM model (P-Bias = -0.0261%) and the CEEMDAN-LMD-NN model (P-Bias = -0.0171%). This finding is consistent with the RMSE values, where the single-phase NN model has an RMSE value of 0.8708°*C*, while the CEEMDAN-ELM and CEEMDAN-LMD-NN models have RMSE values of 1.2172°*C* and 0.9521°*C*, respectively. This indicates that the single-phase NN model is equally optimal for a long-term temperature prediction just like the three-phase CEEMDAN-LMD-NN mode.

From the analysis, the three-phase CEEMDAN-LMD-NN prediction model indicates the propensity of employing such a composite model for short-term (2-months), medium-term (4-months), long-term (6-months) prediction of temperature with an acceptable degree of precision (Table 8). In fact, the superior performance of the CEEMDAN-LMD-NN model presented in this study is consistent with the study of [40] that demonstrated the superiority of the three-phase model over the two-phase and single-phase models in terms of runoff prediction.

**Table 8:** The 2-step, 4-step, 6-step-ahead prediction performance comparison of the temperature data for different three-phase prediction models.

Model	Upper East				
	RMSE (°*C*)	MAPE (%)	MAE (°*C*)	P-Bias(%)	RSR
*2-month ahead*					
ARIMA	0.3746	0.0134	0.3634	**0.0032**	0.7461
CEEMDAN-ARIMA	0.3995	0.0157	0.4512	-0.0057	0.7974
CEEMDAN-LMD-NN	**0.3394**	**0.0115**	**0.3111**	0.0048	**0.6760**
*4-month ahead*					
NN	0.6487	0.0183	0.5122	0.0179	0.5900
CEEMDAN-ARIMA	0.3923	0.0121	0.4668	**-0.0023**	0.3749
CEEMDAN-LMD-NN	**0.3579**	**0.0110**	**0.3371**	-0.0039	**0.3255**
*6-month ahead*					
NN	**0.8708**	0.0271	0.7381	**-0.0029**	**0.7606**
CEEMDAN-NN	1.2172	0.0292	0.7764	-0.0261	1.0632
CEEMDAN-LMD-NN	0.9521	**0.0225**	**0.6027**	-0.0171	0.8316

For the single-phase, two-phase, and three-phase cases, the constructed ELM model and its hybrid models performed poorly when predicting temperature in all the lead times. The standalone ARIMA and its hybrid two-phase and three-phase models performed better compared to the ELM and its associated hybrid models. This result is contrary to previous results in several other studies [41, 42] where the ARIMA model has been applied. Generally, the NN and its hybrid models were the best performing models in relation to the ELM and ARIMA. However, the hybridization of the LMD algorithm contributed to the optimal performance of the NN model integrated with the CEEMDAN algorithm. The optimal performance of the LMD algorithm is consistent with different studies.

It is important to note that the prediction errors for the short term (2-months ahead) and the meidum term (4-months ahead) are low as compared to the long-term (6-months ahead) prediction. However, generally, based on the range of prediction errors, it can be stated that the CEEMDAN-LMD-NN model is an optimal choice for long-term prediction of temperature for weather index insurance/weather derivatives ratemaking and pricing.

## Conclusion and recommendation

In this paper, we propose a three-phase hybrid prediction model that combines the robustness of CEEMDAN-LMD with the NN algorithm to improve prediction accuracy. We applied the resulting model, CEEMDAN-LMD-NN, to the Upper East region of Ghana to forecast 2-, 4-, and 6-month-ahead temperature predictions, and benchmarked it against standalone (ARIMA, ELM), two-phase hybrid models (CEEMDAN-ARIMA, CEEMDAN-ELM, CEEMDAN-NN), and three-phase hybrid models (CEEMDAN-LMD-ARIMA, CEEMDAN-LMD-ELM). In comparison to the standalone, two-phase, and other three-phase hybrid models, the CEEMDAN-LMD-NN model demonstrated greater precision and stability, although model error increased with increasing lead time (i.e., 4- and 6-month forecasts). Based on this evidence, we conclude that the three phase hybrid CEEMDAN-LMD-NN model coupled with CEEMDAN and LMD decomposition methods implemented in an NN model effectively removes non-stationary, non-linear, and volatile characteristics associated with original time series data, which makes it difficult for standalone statistical and machine learning models to map the underlying complexity in the antecedent temperature data used as the model inputs. A thorough analysis of forecasted and observed data sets foresaw the exceptional superiority of the three-phase hybrid CEEMDAN-LMD-NN.

Despite the three-phase hybrid CEEMDAN-LMD-NN model’s optimal performance in comparison to its counterpart models for multi-step temperature prediction, the study has limitations that open the door to future investigation. Combining the three-phase hybrid model with a feature selection tool that uses a lot of satellite and observationally modelled atmospheric products could help with temperature forecasting in places like Ghana that don’t have a lot of data. Additionally, the market for weather index insurance may value forecast information due to the demand for pre- and post-forecast products. In a future study, we propose to use weather index insurance premiums as features for weather prediction models.
